# Application of Mesenchymal Stem Cells in Female Infertility Treatment: Protocols and Preliminary Results

**DOI:** 10.3390/life14091161

**Published:** 2024-09-13

**Authors:** Sofia Chatzianagnosti, Iasonas Dermitzakis, Paschalis Theotokis, Eleni Kousta, George Mastorakos, Maria Eleni Manthou

**Affiliations:** 1 School of Medicine, National and Kapodistrian University of Athens, 11527 Athens, Greece; sofiachatzianagnosti@gmail.com (S.C.); lkousta@gmail.com (E.K.); 2 Department of Histology-Embryology, School of Medicine, Aristotle University of Thessaloniki, 54124 Thessaloniki, Greece; ptheotokis@auth.gr (P.T.); mmanthou@auth.gr (M.E.M.); 3 Department of Endocrinology, Diabetes Mellitus and Metabolism, Aretaieion Hospital, Medical School, National and Kapodistrian University of Athens, 11527 Athens, Greece; mastorakg@gmail.com

**Keywords:** molecular techniques, mesenchymal stem cells, stem cell therapy, signaling pathways, reproductive system, infertility treatment

## Abstract

Infertility is a global phenomenon that impacts people of both the male and the female sex; it is related to multiple factors affecting an individual’s overall systemic health. Recently, investigators have been using mesenchymal stem cell (MSC) therapy for female-fertility-related disorders such as polycystic ovarian syndrome (PCOS), premature ovarian failure (POF), endometriosis, preeclampsia, and Asherman syndrome (AS). Studies have shown promising results, indicating that MSCs can enhance ovarian function and restore fertility for affected individuals. Due to their regenerative effects and their participation in several paracrine pathways, MSCs can improve the fertility outcome. However, their beneficial effects are dependent on the methodologies and materials used from isolation to reimplantation. In this review, we provide an overview of the protocols and methods used in applications of MSCs. Moreover, we summarize the findings of published preclinical studies on infertility treatments and discuss the multiple properties of these studies, depending on the isolation source of the MSCs used.

## 1. Introduction

Infertility is characterized by the inability to achieve pregnancy, while trying to, for at least 12 months, including the occurrence of miscarriages [1,2]. This condition affects 48 million reproductive-age couples globally, with men and women each contributing 40% of cases, while the remaining 20% are due to both partners or unexplained causes [3,4]. More specifically, the most common causes of subfertility include the following: male factor subfertility (30%), such as defects during spermatogenesis; ovulatory dysfunction (25%), such as polycystic ovary syndrome (PCOS); hypothalamic dysfunction, primary ovarian insufficiency (POI), or tubal issues (20%); uterine or peritoneal disorders (10%) [1,5,6,7]. Age-related factors, infections, and environmental and lifestyle factors such as smoking or obesity may also contribute to subfertility. Additionally, medical treatments like surgery, radiotherapy, or chemotherapy for severe diseases such as cancer can compromise fertility [8,9,10,11,12,13].

Numerous molecular factors that are involved in these reproductive disorders reflect an individual’s overall systemic health, to the same extent as the complexity of related signaling pathways [14,15,16]. The lack of specific biomarkers and knowledge of the mechanisms involved in these conditions increases the gap between successful diagnoses and effective treatments, indicating a need for extended research.

Currently, stem cell therapy, particularly mesenchymal stem cell (MSC) therapy, is emerging as a promising treatment for subfertility conditions such as PCOS, POI, endometriosis, Asherman syndrome, and anovulation after cancer treatment. The ability of MSCs to self-renew and differentiate into multiple cell types, along with their anti-inflammatory and proangiogenic effects, make them potential candidates for enhancing pregnancy outcomes [17,18,19,20,21,22]. However, extensive research is still required to fully understand stem cell biology and optimize clinical applications.

In this review, we discuss the current status, existing biological mechanisms, and clinical applications of MSC therapies in patients facing infertility-related issues. We mention their properties, which emerge from their source of isolation; additionally, we discuss the protocols, methods, and molecular techniques employed for MSCs’ applications in clinical practice. This information may be useful to investigators working on MSC therapies for restoring fertility.

## 2. Stem Cell Therapy and the Role of MSCs

So far, people wishing to conceive who are facing infertility issues will either receive medication, undergo surgical procedures, or undergo cryopreservation treatments in combination with assisted reproduction technologies in an effort to achieve a positive pregnancy outcome [3,14,23,24]. However, many complications, such as multifetal gestations, ectopic pregnancy, ovarian hyperstimulation syndrome, ischemic injury limitations, birth defects, high risk of breast and ovarian cancer, and vein thromboembolism, are associated with the therapeutic methods discussed in [3,8,14,24]. Investigators have indicated that the overall infertility rate remains greater than 80% [14,25]. Developing an effective therapeutic approach requires significant consideration of physical, psychological, financial, and time-related factors. Additionally, it is essential to utilize new cellular-level technologies to aid in building a full understanding of underlying molecular mechanisms [14].

Stem cells are defined as cells which remain in an undifferentiated form in embryos and adult tissues and which have a self-renewal ability and a differentiation ability in multiple mature cell types under certain conditions [3,24,26,27]. Thus, they can contribute to an organ’s damage repair and restoration level. They are classified, according to their origin, as embryonic stem cells (ESCs), adult stem cells (including mesenchymal stem cells, MSCs), induced pluripotent stem cells (iPSCs), spermatogonial stem cells (SSCs), and ovarian stem cells [24,28,29]. However, ESC therapies face ethical and controversial concerns regarding potential tumor formation, lack of functionality, immune rejection, and inefficiency after transplantation [3,6,30,31]. Moreover, their difficult and demanding manipulation protocols, which include the handling of early-stage embryos, have led to religious and political concerns, to the same extent as the induction of abortion. Therefore, distinct policies and ethical regulations are necessary if they are to become therapeutically applicable [6,32,33].

Mesenchymal stem cells (MSCs) have received increasing attention for their potential applications in several diseases; this is particularly the case for fertility-related disorders, as they can restore ovarian function [3,7,15] due to their easy in vitro isolation protocols and culture handling, which have not led to major ethical concerns [7,8]. MSCs are spindle-shaped somatic cells with a stromal origin; they can be extracted from several sources, such as bone marrow, adipose tissue, menstrual blood, umbilical cords, amniotic fluid, placental tissue, the endometrium, and other tissues or organs [1,8,15,34]. Moreover, they can modulate immune responses and proliferate, differentiate, self-renew, and interact through cell–cell signaling; most importantly, they secrete paracrine growth factors, such as chemokines, cytokines, miRNAs, and extracellular vesicles, resulting in tissue regeneration and repair [6,14,24]. Specifically, with reference to infertility treatments, their most prominent features include their ability to differentiate and their secretory, immunomodulatory, and anti-inflammatory capacity; additionally, they can perform mitochondrial transfer following migration to injured tissues [3].

### 2.1. Protocols for Cultures of MSCs and Therapeutic Methods Employing MSCs

Mesenchymal stem cells can be categorized based on their source: bone marrow MSCs—BM-MSCs; umbilical cord stem cells—UC-MSCs; amniotic fluid mesenchymal stem cells—AF-MSCs; menstrual stem cells—Men-MSCs; adipose-derived stem cells—AD-MSCs; placenta-derived stem cells—PMSCs [3,35]. They have been found to show efficacy in treating fertility-related disorders in both preclinical and clinical studies [3]. Their effective differentiation and proliferation levels are linked to various parameters, such as the age of a donor, the culture conditions, the isolation methodology, the materials employed, the route of cell expansion, the route of administration, the cell dosage, and the microenvironment of the source from which a sample of MSCs has been extracted [8,36,37].

The Mesenchymal and Tissue Stem Cell Committee of the International Society for Cellular Therapy introduced specific criteria for MSCs used in research and clinical practice [7,15,38]. Firstly, MSCs should be plastic-adherent when preserved in standard cell culture media and be able to differentiate, under certain conditions, into osteoblasts, adipocytes, and chondroblasts [15,39]. Secondly, they express specific cell surface molecules, CD73, CD90, and CD105; but they do not express certain hemopoietic markers, i.e., CD11b, CD14, CD19, CD34, CD45, CD79a, and HLA-DR [7,40,41]. Despite the common criteria established, there is no universal agreement on the optimal source and method for MSC isolation, purification, culture, or the animal model that should be used for their extraction [42,43,44,45].

Isolation and culture protocols vary depending on the source of MSCs and involve specific methodologies that are tailored to each tissue type. Solid tissue sources like bone marrow require density centrifugation or enzymatic digestion, while blood or fluid samples undergo simpler centrifugation methods [46]. Adherence properties aid in MSC isolation, while contamination with hematopoietic stem cells necessitates sorting techniques, such as magnetic bead sorting or fluorescence-activated cell sorting (FACS) [46].

The in vitro culture of cells is an essential requirement in the expansion and multiplication of the number of MSCs, enabling a sufficient quantity to be reached so they can be used effectively in medical treatment. Culture conditions, including medium composition and incubation parameters, influence proliferation and differentiation potential. For instance, MSCs are typically cultured in Dulbecco’s Modified Eagle Medium (DMEM) or Minimum Essential Medium (MEM), supplemented with fetal bovine serum (FBS) or human platelet lysate. L-DMEM, DMEM-F12, and LG-DMEM are also being used [8,9,47,48,49,50], with most methods containing glucose and ions like magnesium, calcium, potassium, sodium, and phosphate, and amino acids [46,51]. While monolayer cultures are common due to their cost-effectiveness, 3D static cultures and bioreactors offer enhanced cell proliferation and differentiation capabilities, mimicking in vivo conditions with minimal stress [46,52,53,54,55]. These scaled-up cell manufacturing systems overcome conventional methods in terms of minimized media exposure and manufacturing time, as well as anticontamination and labor costs [37,56,57].

Quality control during MSC expansion adheres to Good Manufacturing Practices (GMPs), ensuring standardized production. Growth factors such as fibroblast growth factors (FGFs) and platelet-derived growth factor BB (PDGF-BB) may enhance cell yield and viability [44,45]. Long-term culture approaches present certain challenges, such as reduced potency and senescence, which are mitigated by hypoxic conditions [37,58].

Characterization techniques include flow cytometry for surface marker analysis, tri-lineage differentiation assays, gene expression profiling, and functional assays, such as colony-forming unit assays (CFU-Fs). Delivery methods vary based on the therapeutic target in question, with intravenous, intra-arterial, or local injections and surgical implantations being common approaches [38,59,60,61,62,63].

After the ex vivo isolation and expansion of MSCs, the next critical step for clinical application is to effectively deliver fresh cells to the target site. Common delivery methods include the following: intravenous injection for systemic delivery, which is often used in treating inflammatory or autoimmune conditions; intra-arterial or local injection, for targeted delivery to specific organs or tissues; surgical implantation, in which MSCs are embedded in scaffolds [46]. Scaffolds consist of either substances found in the body, such as collagen [35], or synthetic substances; scaffolds can be considered to be vehicles for tissue regeneration. They can mimic the native characteristics of tissue and provide vasculogenesis, cell migration, and the attachment of the MSCs [46].

In order to manage the homing of the stem cells and understand their regenerative ability, several bioactive materials are tagged to the cells, providing a way of tracking them and guiding them in the right direction. PKH26, green fluorescent protein (GFP), F-fluorodeoxyglucose, enhanced green fluorescent protein (EGFP), CM-Dil, DiIC fluorescence dyes, molday ion rhodamine B, and iron oxide particles are the substances that are used more frequently in immunohistochemistry and fluorescence microscopy [7,15,18,19,20,62,63,64,65,66,67,68,69]. Moreover, additional methods include magnetic resonance imaging (MRI), in which MSCs are labelled with iron oxide nanoparticles and provide high-resolution images, bioluminescence imaging (BLI) using luciferase expression, positron emission tomography (PET), single-photon emission-computed tomography (SPECT), and computed tomography (CT) [64,65,66,70,71,72].

Storage and transportation protocols are critical in maintaining MSC viability and functionality. Cryopreservation in liquid nitrogen or short-term refrigeration with cryoprotectants ensures cell stability during transit, though challenges like cell viability and apoptosis rates remain [37,67,68]. Regarding the short-term storage of MSCs, the cells can be preserved at 4 °C for 72 h before transplantation using cryoprotectant mediums such as DMSO. However, it is important to consider that frozen transportation can lead to a substantial reduction in viable cells and an increase in apoptotic and senescent cells. For non-frozen transportation, temperature preservation at 37 °C and cell metabolism maintenance is challenging. Additionally, non-frozen methods are inconvenient due to packaging limitations for long-distance transport [37,67,73].

Lastly, regarding the long-term storage of MSCs, stem cell banking aims to store the maximum number of samples cost-effectively. Cryovials and cryobags are available in various sizes. It is crucial that stem cell banks are able to maintain quality control, ensuring the high quantity and quality of cryopreserved samples [37,69,73].

### 2.2. Biology of MSCs

The therapeutic properties of MSCs in fertility-related diseases look very promising in cell therapy treatments. However, controversies and concerns surrounding their outcomes in clinical practice remain in the spotlight; this is because there is a lack of knowledge on the function of MSCs at the cellular and molecular levels [6,8,14]. Thus, developing an effective therapeutic approach requires cellular-level technologies to fully understand the underlying molecular mechanisms. These mechanisms are described further on in this paper.

**Figure 1:**
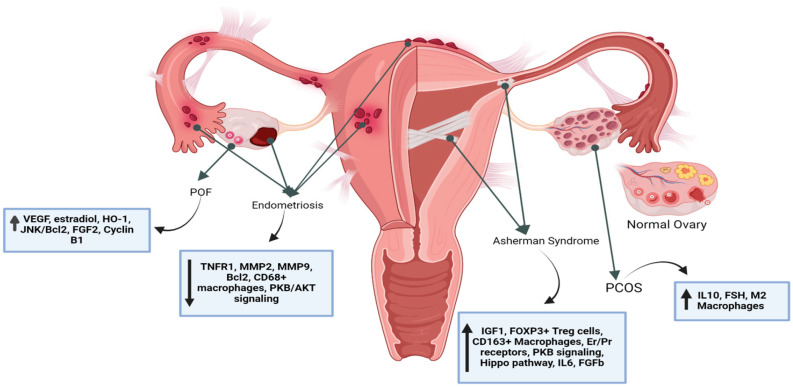
Simplified overview of female reproductive disorders (Asherman syndrome, polycystic ovarian syndrome (PCOS), premature ovarian insufficiency (POI), and endometriosis) in the internal reproductive female organs, as well as the healing effects of mesenchymal stem cells (MSCs).

Mesenchymal stem cells can regulate immune response, inflammation, angiogenesis, and oxidative stress in disorders, causing infertility through mechanisms and signaling pathways which alter cell differentiation or contribute to the secretion of several transcription factors that are essential for tissue restoration and improvement (Figure 1) [1,7,15,24].

Through the NF-kB/Rap1 pathway (a telomere-associated protein), MSCs can regulate immune responses and inflammation [7,15,74] and delay the pro-inflammatory function of the target tissue by increasing Treg production in multiple ways [7,75]. Firstly, they can modify the cytokine profile of the dendritic cells that are responsible for initiating every antigen-specific immune response [7,75]; also, they can convert T cells to Treg through the transformation of M1 macrophages into M2 macrophages, resulting in tissue repair and healing [3,76,77]. Secondly, MSCs can reduce the levels of IL-6 and IL-1β that are secreted from macrophages, thus increasing the number of Treg cells [7,75,78]. Furthermore, they are able to limit inflammation by secreting multiple factors such as TGFβ, HGF, lipoxinA4, TNFα, PGE2, IDO, and ΝO, thus leading to increased levels of Τreg cells by upregulating the transcription of FOXP3/CTLA4/GITR genes [7,79,80,81,82]. In addition, MSCs can preserve immune tolerance by suppressing the differentiation of Th17 through the inhibition of IFNγ production and the alteration of the response of Th1 to Th2 [7,75,78]; moreover, they can decrease immune rejection through the downregulation of the MHC-II reaction [35,83].

The differentiation capacity of MSCs is also essential for restoring infertility. They can differentiate into epithelial, stromal, and endothelial cells and enhance the recovery of ovarian function [1,3,7]. However, the number of MSCs that can differentiate and be integrated functionally is too small, inhibiting observation of significant improvements [1,3,7]. For instance, BM-MSCs have the ability to differentiate into granulosa and endometrial cells [15,84], but their long replication cycle decreases the total number of their differentiated population [15,85]. The exact mechanism by which MSCs differentiate into target cells, such as oocytes or supporting cells, after migrating to injured tissues, remains unclear [1,3,7]. Thus, it has been indicated that the increase in ovarian function is mediated by the paracrine effects of MSCs and their secretory capacity [1,3,7].

Nowadays, researchers are suggesting that the beneficial effects of MSCs in reproductive treatments are connected to various bioactive secretory factors, including insulin-like growth factor (IGF), vascular endothelial growth factor (VEGF), and several cytokines [1,6,15,24,86]. Through their secretome, MSCs are able to restore tissue cellular composition by regulating the immune response, stimulating angiogenesis, and maintaining the viability of the microenvironment [3,24,86,87]. More specifically, MSCs, by secreting VEGF, which binds to its receptor (VEGFR) on endothelial cells, activate the PI3K/Akt signaling pathway; this is a critical pathway for promoting angiogenesis and enhancing cell survival [15]. Moreover, MSCs secrete TGF-β, which engages the TGF-β receptors on target cells. This activation leads to the phosphorylation of Smad proteins, which then translocate to the nucleus to regulate the expression of genes involved in immune modulation and tissue repair [88].

Moreover, the anti-apoptotic capacities of MSCs seem to be advantageous in patients facing infertility issues due to previous or current cancer therapy treatment [8,89]. These patients present increased expression levels of the p21 gene, which causes cell arrest in the G1/S or G2/M phase and low expression levels of G2 cyclin, which enhances the proliferation of granulosa cells [8,47]. Mesenchymal stem cells can increase G2 cyclin and decrease the transcription of the p21 gene through the downregulation of p53 and Bax genes [8,47]. Also, through their secretome, they can prevent the apoptosis of ovarian follicles [11,90,91] and contribute to resistance to oxidative stress through the upregulation of the Bcl2 anti-apoptotic protein [8,9,34]. In addition, MSCs can modulate anti-oxidative mechanisms by increasing heme oxygenase 1 factor, which participates in inflammation [7,92,93]. In the next section and the table below (Table 1), the multiple properties of MSCs, regarding their isolation source, are presented depending on their application in fertility-related disorders, such as PCOS, POF, preeclampsia, Asherman syndrome, endometriosis, and chemotherapy-induced infertility.

#### 2.2.1. Bone Marrow MSCs

Bone marrow MSCs were first described by Owen and Friedenstein, who isolated them from nucleated bone marrow cells in 1988 [15,94]. They are an important source of multipotent stem cells because of their easy isolation and proliferation in vitro and their ability to migrate effectively to damaged tissue [35]. Thus, they serve as a standard for the comparison of MSCs derived from different sources [15,85]. They can differentiate into chondroblasts, osteoblasts, and adipocytes [15,85], but findings have shown that they can also differentiate into endometrial, endothelial, and granulosa cells [15,84]. Preclinical studies in rats indicate that BM-MSCs can increase endometrial thickness and improve receptivity and lining in rats with endometrial cavity fibrosis [17,35,95]. In addition to that, animal studies have shown that they can induce proliferation and differentiation in the microvascular endothelium of the endometrium through paracrine factors secretion and they can lead to enhanced receptivity in mouse models [35,95,96,97]. Moreover, in women facing infertility issues due to cancer therapy, investigators have shown that BM-MSCs are able to restore the levels of ovarian hormones and folliculogenesis after experimentation in a POF–chemotherapy model [10,35]. Additionally, in a clinical trial focusing on women with POF using BM-MSCs, researchers revealed that the patients showed hormonal improvement, resumption of menses, and an increased pregnancy rate [8]. However, using BM-MSCs as a source of extraction remains challenging due to their invasive isolation method and their ability to differentiate into undesirable cell types, with increasing donor age emerging as a primary motivator in the need for extended research [35].

#### 2.2.2. Umbilical Cord MSCs

Umbilical cord MSCs have increased differentiation and proliferation levels, appear to have low immunogenicity, and show an extended survival time after transplantation [35,98]. They can improve ovarian damage in infertility disorders through three major signaling pathways: MAPK/ERK, insulin signaling, and the G-protein coupled receptor pathway (GPCR) [14,15,99,100]. Through their regulation, UC-MSCs control proliferation, differentiation, and cell death in eukaryotes; moreover, they can modulate cell growth and development [101,102].

Umbilical cord MSCs are able to overturn apoptosis in ovarian cells through two distinct ways. They can either adjust the surface epithelium of the ovaries and the tunica albuginea, a layer of the ovarian surface, or increase the levels of TGFβ factor and CK8/18, enhance proliferation of the cell nuclear antigen, and limit the level of Caspase 3 apoptotic protein [14,103]. At the same time, another clinical study using UC-MSCs loaded in collagen scaffolds demonstrated that they could restore endometrial differentiation, vascularization, and proliferation through the enhancement of the levels of the ERα and angiogenic factors [14,104]. Furthermore, researchers in a phase 2 clinical trial of women with POF demonstrated that the patients appeared to have increased ovarian volume and an increased pregnancy rate following the administration of human UC-MSCs [8]. In addition, angiogenesis could be enhanced through the secretion of several factors from UC-MSCs, such as the placental growth factor, VGF, TGFβ, HGF and anti-inflammatory vascular markers, leading to decreased fibrosis and ovarian restoration [14]. Also, they have been shown to modulate the apoptosis of ovarian cells and help in the restoration of the ovary in both preclinical and human trials [14,49,90,105]. However, limitations of UC-MSC application, such as their high heterogeneity, their low isolation efficiency, and their limited collection at birth, remain challenging [35].

**Table 1:** Overview of preclinical studies using MSCs as therapy for patients facing infertility issues.

MSCs Source	Infertility Disorder	Animal Model	Biological Mechanism	Treatment Outcome	References
BM-MSCs	PCOS	Intraovarian injection in mouse	↑ IL10	↓ Carbs inflammation, steroidogenic gene expression leading to fertility recovery	[7,106]
BM-MSCs	PCOS	Mouse	↑ FSH, ↓ LH, testosterone, MDA levels	↑ Folliculogenesis, oocyte quality, ↓ apoptosis, oxidative stress, inflammation	[7,14,107]
BM-MSCs	PCOS	Intraovarian injection in mouse	↓ CYP17A1/DENNDIA gene expression, BMP2, suppression H295R	↓ Androgen genes, ↑ apoptosis	[7,14,107]
BM-MSCs	Endometriosis	Transplantation in mouse	↓TNFR1 expression	↑ Folliculogenesis, graafian follicle count, ↓ apoptosis in granulosa cells	[7,108,109]
BM-MSCs (CD133+)	AS	Rat uterus	↑ IGF1, Thrombospondin 1 levels/IL10, ↑ FOXP3+ Treg cells/CD163+ M2 macrophages, ↓ CD8+ cytotoxic T cells	↑ Proliferation of endometrial cells around vessels leading to a pro-regenerative environment in which angiogenesis, fibrosis, receptivity, and regeneration of the endometrium are controlled	[7,110,111]
BM-MSCs (PROM1/CD133+)	AS	Women	↑ Er and Pr receptors	↑ Endometrial vascular density and improved menstrual cycle	[35,112]
BM-MSCs	Infertility after chemotherapy	Juvenile macaques	-	↓ Apoptosis, fibrosis, ovarian age, and regeneration of blood vessels and follicles	[14,113]
BM-MSCs	POF after chemotherapy	POF–cyclophosphamide-induced rabbits	↑ VEGF, estradiol, ↓ FSH, apoptotic factor Caspase 3	↑ Ovarian function and restored ovarian structure	[1,15,114]
BM-MSCs	Infertility after chemotherapy	Cisplatin-induced rat	↓ Apoptosis in granulosa cells	Improved perimenopause	[35,115]
BM-MSCs	POF	Women	-	Improved follicular function, menstrual cycle, pregnancy rate, FSH levels, and endometrial thickness	[8]
UC-MSCs	POF	Mice	Regulation of JNK/Bcl2 pathway, ↑ HO-1, regulation of autophagy	Ovarian restoration and ↑ of CD8/CD28 T cells	[7,116]
UC-MSCs	POF	Mouse	Change in the ratio Th1/Th2 cytokines, ↑ HOXA10 gene, ↑ E2, Pr, IL-4, ↓ FSH, IFNγ, IL-2	Improved implantation, ↓ apoptosis of granulosa cells	[7,117,118]
UC-MSCs	Preeclampsia	Endotoxic-induced preeclampsia rat	↓ TNFa/IL-1β, ↑ IL-10	↓ Blood pressure, urine protein, and white cells	[7,119]
UC-MSCs	PCOS	DHEA-induced mice	↓ M1 macrophages, neutrophils, B-lymphocytes, TNFa, IL-1β, IFNγ, fibrosis-related genes (CTGF) transcription, ↑ M2 macrophages	Regulation of inflammation, improved ovarian function and recovery	[7,15,120]
UC-MSCs	Preeclampsia	LPS-induced rat	↑ PPARγ, laminin receptor 1, ↓ MMP2/MMP9/ICAM1	Improved hypertension and fetal weight	[7,121]
UC-MSCs	Preeclampsia	AT1-AA induced hypertension rat	Remodeling of the spiral artery, ↓ injury in the kidney, ↑ placental, mesometrial triangle HO-1 expression	↑ Pregnancy outcome, change in the cytokine profile of the animal	[7,122]
UC-MSCs	Preeclampsia	Th1-induced preeclampsia mouse	↓ TNFa in uterine and splenic lymphocytes	↓ Blood pressure, proteinuria, glomerulonephritis, ↑ fetal–placental growth	[7,123]
hUC-MSCs (Wharton jelly)	Uterine scars	Transplantation with collagen scaffolds in rat	↑ MMP9	Endometrial renewal	[35,124,125]
hUC-MSCs (Wharton jelly)	Endometriosis	Endometrial cells in vitro	↓ MMP2, MMP9, BAC, SMAC, survivin, Bcl2 proteins	↓ Viability, development, invasion, migration of the cells, ↑ their apoptotic activity	[7,126]
hUC-MCSs	AS	Injection with collagen scaffolds in humans	↓ ERa, Vimentin, von Willebrand factor, Ki67	Recovery of uterine adhesions, ↑ cell proliferation, differentiation	[15,113]
UC-MSCs	Age-related infertility	Perimenopausal rats	Cytokines release	Restoration of total follicle count	[14,100]
UC-MSCs	Age-related infertility	-	Phosphorylation of FOXO1 and FOXO3a	Primordial follicles activation	[15,127,128]
hUC-MSCs	POF	Women	-	Improved the development of the number of antral follicles, pregnancy rate, ↑ AMH, E2, FSH levels	[8]
AD-MSCs	Endometriosis	Rats	↓ CD68+ macrophages and pro-inflammatory cytokines	↓ Endometriosis-related inflammation	[7,129]
AD-MSCs	POF	Injection with collagen scaffolds rat	-	↑ Preservation of ADMSCs in ovaries of the rat	[15,48]
AD-MSCs		Rat	↑ VEGF	↑ Angiogenesis and ovarian graft quality	[15,130]
AD-MSCs	POF	Chemotherapy POF-induced mice, rats	Altering gene expression and paracrine cytokines secretion	Improve ovarian function after chemotherapy, ↑ follicle number, oocyte number, corpora lutea	[11,35,131]
Fetal liver MSCs	POF	Mouse	MT1 target, ↑ oxidate protection, Caspase 3, Caspase 9, Bcl2 suppression	↑ Proliferation of granulosa cells, anti-apoptotic effects, ↓ ovarian injury, oxidate damage	[7,132]
Men-SCs	POF	Mice	↑ FGF2	Restoration of ovarian function and structure through ↓ fibrosis, ↓ granulosa cell apoptosis, ↑ follicle counts, normal sex hormones	[7,12,15]
En-MSCs	POF	Mouse, rat, human	↓ Growth arrest, GADD45B factor, ↑ CDC2 and Cyclin B1	Restoration of ovarian function, ↓ granulosa cell apoptosis	[15,133,134,135]
Men-SCs	AS	Rats, rodents	Regulation of PKB signaling	↑ Angiogenesis, immunomodulation, rate of implanted embryos	[7,14,136,137]
Chorionic villous MSCs (CV-MSCs)	Preeclampsia	In vitro	↑ LC3BII through JAK2/STAT3 pathway	↑ Proliferation, invasion, autophagy of trophoblastic cells	[7,138]
Men-SCs	AS	Rat	Regulation of Wnt5a and Gdf5 factors and Hippo pathway	↑ Endometrial growth, improve endometrial proliferation and angiogenesis, ↓ fibrosis, inflammation	[1,15,139]
Men-SCs	Endometrial damage	Mouse	↑ Keratin, vimentin, VEGF, ↓ DNA damaging factors, PKB/AKT signaling modulation	↑ Rate of embryo transplantation	[14,17,35,137]
A-MSCs	Age-related infertility	Mouse	Regulation of PRKAA2/AMPK/FOXO3/FOXO3A pathway	↑ Ovarian function and oocyte maturation	[35,140]
A-MSCs	AS	Rat	↓ TNFa and IL1b, ↑ IL6 and FGFb	Ovarian restoration, ↓ inflammation	[35,141]
P-MSCs	-	Rat	Secretion of KIT ligand (KITLG/SCF) result to ↑ Lin28a, Lhx8, Nanos3, Nobox genes expressions	Improve ovarian function	[35,98]
P-MSCs	-	Ovariectomized rats	↑ Estrogen and folliculogenesis-related genes expression	Improve ovarian function	[15,142]

#### 2.2.3. Adipose Tissue MSCs

Adipose tissue MSCs are easy to isolate in large quantities from adipose tissue compared to bone marrow aspiration; the process involves a relatively simple and minimally invasive liposuction procedure [1,35,143]. Thus, they could have potential in cell therapy in the near future. More specifically, findings have shown that their functions could be very beneficial in Asherman syndrome treatment. Preclinical studies in Asherman syndrome rats showed that AD-MSCs combined with estrogen therapy decrease inflammation, improve endometrial regeneration, and enhance endometrial thickness [7,15,35,144]. In mammals, AD-MSCs appear to have same results in combination with induction of endometrial glands and microvessels [35,145,146]. Furthermore, in a clinical trial of women with POF, investigators showed that the administration of AD-MSCs improved the antral follicle diameter [8]. In addition, another study revealed that using both AD-MSCs and BM-MSCs can improve the injured endometrium by diminishing collagen deposition and fibrosis [7,147]. Moreover, they suggested that the intrauterine injection of AD-MSCs leads to a thicker endometrium than intravenous injection of BM-MSCs [7,147] does, indicating a preference for AD-MSCs application in cell therapy. However, AD-MSCs exist within a complex environment and interact with various other factors and cells. When these cells are isolated through liposuction and separated from their original niche, their characteristics, such as their ability to proliferate, may diminish [35,148]. Thus, further investigation needs to be performed to establish their efficient applications as an effective source of MSCs.

#### 2.2.4. Menstrual-Blood-Derived MSCs/Endometrial MSCs

Menstrual-blood-derived endometrial SCs are similar to endometrial stem cells. They express both MSCs and ESCs markers and have greater proliferative and regenerative ability than the BM-MSCs or AD-MSCs [35,149,150]. Moreover, their easy, non-invasive isolation method and the fact that they do not present an immune rejection risk makes them a good candidate for infertility treatment [14,151,152]. Menstrual blood MSCs in combination with Er/Pr therapy have been shown to differentiate into endometrial cells and create endometrial tissue in a mouse model [15,153]. Moreover, they have been shown to be able to improve endometrial structure and increase endometrial thickness in an Asherman syndrome model [15,154]. Another preclinical study reveals that En-MSCs could repair the injured endometrium through a decrease in fibrosis and inflammation [35,139,155].

#### 2.2.5. Amniotic Fluid Stem Cells/Amniotic Epithelial Stem Cells

Amniotic fluid SCs appear to have an increased differentiation level with adipose, muscle, and bone cells; additionally, they are free of the ethical concerns that are present for ESCs [15,156]. Their beneficial applications in infertility treatment seem to be very promising due to their rich secretome. FSHR, VEGF, IGF1, TGFa, BMP4, EGF, and TGFb are some of the factors that they secrete [15,100]. Also, investigators have demonstrated that they can decrease follicular atresia and preserve healthy follicles in patients undergoing chemotherapy, despite the fact that they do not differentiate into granulosa cells [15,157]. Moreover, they have been shown to improve ovarian function and enhance ovarian regeneration in a chemotherapy-induced POF rat model, indicating their future potential in patients with cancer [35,158]. Another preclinical study in Asherman syndrome mouse model showed that amniotic epithelial stem cells (A-ESCs) could increase endometrial stromal cell proliferation, leading to increased levels of angiogenesis, endometrial thickness, autophagy, and decreased fibrosis [35,159].

#### 2.2.6. Placental MSCs

Placental MSCs (PMSCs) are easily accessible through non-invasive techniques; additionally, they are not affected by a donor’s age and they express common BM-MSC markers [35,160,161]. They can secrete various factors such as CSF3/G-CSF, IL6, IL8, IL10, and CCL5/RANTES, thus leading to immunoregulation, self-renewal, and differentiation. These cytokines make them suitable for cell therapy [35,162]. In patients with preeclampsia, PMSCs, through their rich secretome, can modulate signaling pathways involved in this disorder [15,163]; additionally, they can affect the regulators of the G1/S phase cell cycle checkpoint, leading to improvements in symptoms [15,164]. Investigators have indicated that PMSCs could be a promising solution for patients with POF. A preclinical study using POF mice showed that PMSCs could enhance ovarian function by decreasing the levels of FSH, LH, and estradiol and by increasing the levels of FSHR and AMH through the regulation of PI3K/Akt signaling [15,165]. Another study using POF mice demonstrated that PMSCs, through the downregulation of the IRE1 pathway, could reduce apoptosis in granulosa cells and improve ovarian function and structure [15,166].

## 3. Discussion

The high prevalence of reproductive disorders and infertility is unsurprising given the complexity of successful reproduction, which requires functional gonads, sex determination, neuroendocrine competency, and gametogenesis [167]. Disturbances in the central nervous system and hormonal imbalances demonstrate the correlation between infertility and endocrine disorders [14,17,168]. The lack of specific biomarkers and comprehensive knowledge of the mechanisms involved in infertility conditions highlights the gap between successful diagnoses and effective treatments [14,168].

Mesenchymal stem cell therapies offer a promising alternative treatment for various diseases, including infertility-related conditions, due to their easy isolation, handling, and beneficial biological effects [8,14]. Despite progress in research surrounding MSC therapies, several research questions remain unsolved. Key uncertainties include whether the therapeutic benefits of MSCs originate from their differentiation into reproductive cells or their paracrine signaling, which influences the local environment [1,3,15]. The mechanisms by which MSCs modulate the immune environment in the female reproductive system are not fully understood, raising concerns about tumorigenicity, immune reactions, and unintended tissue regeneration [3,14,15,169]. Additionally, the lack of standardized protocols for MSC isolation, expansion, and administration lead to significant variability in study outcomes [8,14,170]. As a result, comparing results between different experiments becomes challenging, and in some cases, it becomes nearly impossible due to the inconsistencies in the protocols used.

Furthermore, the ideal source for MSCs extraction and the impact of donor variability, such as age and health status, remain unclear in clinical practice [42,59,169]. Optimal dosing, dose–response relationships, and effective delivery routes require further exploration in human trials [15,59,170]. Moreover, the efficacy and long-term safety of MSC therapies remain ambiguous [8] for the following reasons: limited data; the absence of personalized treatments based on patient factors, such as genetics or the severity of the infertility condition [14,59]; a lack of data on live birth rates; the scarcity of long-term follow-up with patients undergoing MSC treatments [8,14]. These limitations hamper our ability to compare results and develop universally accepted guidelines, maintaining the difficulty that arises in ensuring the consistency and efficacy of MSC therapies [14,15]. Lastly, ethical concerns and socioeconomic considerations, particularly around donor consent and genetic modifications, remain unresolved, further complicating the adoption of MSC therapies for infertility-related disorders [14].

Therefore, the need for collaboration between investigators and bioethicists to establish ethical guidelines for MSC therapies in humans seems to be crucial. Firstly, investigators should prioritize cost-effectiveness evaluations and develop strategies to enhance the accessibility of MSC treatments, particularly in resource-limited settings. The safety of MSC therapies requires extensive investigation, including larger and more diverse clinical trials in combination with long-term follow-up studies to assess outcomes and potentially delayed adverse effects [8,59]. Future research should focus on standardizing stem cell preparation [170], adhering to GMP guidelines, and addressing international regulatory challenges to ensure consistency and reproducibility [14,15,169]. Optimizing MSC dosage and administration should be tailored to specific diseases, guided by systematic dose–response studies [15]. A deeper understanding of the molecular mechanisms that are related to homeostasis, tissue repair, immune modulation, and MSCs differentiation could improve the efficacy of clinical trials [1,3,15]; advanced techniques like single-cell sequencing and proteomics are recommended for these trials.

Lastly, the potential for combining MSC therapies with other fertility treatments, such as hormonal treatments and in vitro fertilization, should be explored to determine their efficacy and safety compared to standard therapies [3,8,15]. Thus, researchers should foster interdisciplinary collaboration across various fields, such as cell biology, reproductive medicine, immunology, and regenerative medicine [14,15]; this would involve the development of data-sharing platforms, the promotion of cross-disciplinary projects, and the definition of common research objectives to advancing research, enabling the sharing of protocols, and ultimately enhancing transparency and reproducibility in MSC studies [15].

In conclusion, this review provides a summary of the infertility conditions affecting people assigned female at birth, summarizing the signaling pathways, traditional treatment approaches, and diagnostic methods of these conditions. This paper also discusses the current statuses of MSC therapies for patients experiencing infertility-related conditions, examining their biological mechanisms and clinical applications; additionally, the advantages and disadvantages of the approaches are discussed. The knowledge shared here aspires to advance the frontier of fertility restoration, addressing a profound challenge that affects millions of people trying to conceive globally.

## Abbreviations

AD-MSCs	Adipose-derived Mesenchymal Stem Cells
AESCS	Amniotic Epithelial Stem Cells
AF-MSCs	Amniotic Fluid Mesenchymal Stem Cells
AKT	Protein Kinase B
AMH	Anti-Müllerian Hormone
AMPK	AMP-activated Protein Kinase
AS	Asherman Syndrome
AT1-AA	Angiotensin II Type 1 Receptor Autoantibodies
BAC	Bacterial Artificial Chromosome
Bax	Bcl-2-associated X protein
BCL2	B-cell Lymphoma 2
BLI	Bioluminescence Imaging
BM-MSCs	Bone Marrow Mesenchymal Stem Cells
BMP2	Bone Morphogenetic Protein 2
BMP4	Bone Morphogenetic Protein 4
CCL5	C-C Motif Chemokine Ligand 5
CD105	Cluster of Differentiation 105
CD11b	Cluster of Differentiation 11b
CD14	Cluster of Differentiation 14
CD163	Cluster of Differentiation 163
CD19	Cluster of Differentiation 19
CD28	Cluster of Differentiation 28
CD34	Cluster of Differentiation 34
CD45	Cluster of Differentiation 45
CD68	Cluster of Differentiation 68
CD73	Cluster of Differentiation 73
CD79	Cluster of Differentiation 79
CD79a	Cluster of Differentiation 79a
CD8	Cluster of Differentiation 8
CDC2	Cell Division Cycle 2
CFU-F	Colony Forming Unit Fibroblast
CK8/18	Cytokeratin 8/18
CM-Dil	CM-1,1′-dioctadecyl-3,3,3′,3′-tetramethylindocarbocyanine perchlorate
CSF3	Colony-Stimulating Factor 3
CT	Computed Tomography
CTGF	Connective Tissue Growth Factor
CTLA4	Cytotoxic T-Lymphocyte Associated protein 4
CVMSCs	Chorionic Villus Mesenchymal Stem Cells
CYP17A1	Cytochrome P450 17A1
DENNDIA	Differentially Expressed in Normal and Neoplastic Development, Isoform A
DHEA	Dehydroepiandrosterone
DilC	1,1′-dioctadecyl-3,3,3′,3′-tetramethylindocarbocyanine perchlorate—C
DMEM	Dulbecco’s Modified Eagle Medium
DMSO	Dimethyl Sulfoxide
DNA	Deoxyribonucleic Acid
E2	Estradiol
EGFP	Enhanced Green Fluorescent Protein
En-MSCs	Endometrial Mesenchymal Stem Cells
ER	Estrogen Receptor
ERK	Extracellular-signal-Regulated Kinase
ERα	Estrogen Receptor alpha
ESCs	Embryonic Stem Cells
F12-DMEM	Ham’s F-12 Nutrient Mixture supplemented Dulbecco’s Modified Eagle Medium
FACS	Fluorescence-Activated Cells Sorting
FBS	Fetal Bovine Serum
FGF	Fibroblast Growth Factor
FGF2	Fibroblast Growth Factor 2
FGFb	Fibroblast Growth Factor basic
FOXO1	Forkhead Box O1
FOXO3a	Forkhead Box O3a
FOXP3	Forkhead Box P3
FSH	Follicle-Stimulating Hormone
FSHR	Follicle-Stimulating Hormone Receptor
GADD45B	Growth Arrest and DNA-Damage-inducible, beta
G-CSF	Granulocyte Colony-Stimulating Factor
Gdf5	Growth Differentiation Factor 5
GFP	Green Fluorescent Protein
GITR	Glucocorticoid-Induced TNFR-related protein
GMP	Good Manufacturing Practices
GPCR	G Protein-Coupled Receptor
H295R	Human adrenocortical carcinoma cell line (NCI-H295R)
HGF	Hepatocyte Growth Factor
HLA-DR	Human Leukocyte Antigen locus DR
HO-1	Heme Oxygenase 1
HOXA10	Homeobox A10
hUC-MSCs	Human Umbilical Cord Mesenchymal Stem Cells
ICAM1	Intercellular Adhesion Molecule 1
IDO	Indoleamine 2,3-dioxygenase
IFNγ	Interferon gamma
IGF1	Insulin-like Growth Factor 1
IL10	Interleukin 10
IL-1β	Interleukin-1 beta
IL-2	Interleukin 2
IL-4	Interleukin 4
IL6	Interleukin 6
iPSCs	Induced Pluripotent Stem Cells
IRE1	Inositol-Requiring Enzyme 1
JAK2	Janus Kinase 2
JNK	c-Jun N-terminal Kinase
KITLG	KIT Ligand (also known as Stem Cell Factor, SCF)
LC3BII	Microtubule-associated protein 1A/1B-light chain 3B, form II
L-DMEM	L-glutamine-supplemented Dulbecco’s Modified Eagle Medium
LG-DMEM	Low Glucose Dulbecco’s Modified Eagle Medium
LH	Luteinizing Hormone
Lhx8	LIM Homeobox 8
Lin28a	Lin-28 Homolog A
LPS	Lipopolysaccharide
MAPK	Mitogen-Activated Protein Kinase
MDA	Malondialdehyde
MEM	Minimum Essential Medium
Men-MSCs	Menstrual Mesenchymal Stem Cells
MHC-II	Major Histocompatibility Complex class II
miRNAs	Micro Ribonucleic Acid
MMP2	Matrix Metalloproteinase 2
MMP9	Matrix Metalloproteinase 9
MRI	Magnetic Resonance Imaging
MSCs	Mesenchymal Stem Cells
MT1	Metallothionein 1
Nanos3	Nanos Homolog 3
NF-kB	Nuclear Factor kappa-light-chain-enhancer of activated B cells
NO	Nitric Oxide
Nobox	Newborn Ovary Homeobox
p21	Cyclin-dependent kinase inhibitor 1 or CDKN1A
p53	Tumor Protein p53
PCOS	Polycystic Ovarian Syndrome
PDGF-BB	Platelet-Derived Growth Factor—BB
PET	Positron Emission Tomography
PGE2	Prostaglandin E2
PI3K	Phosphoinositide 3-Kinase
PKB	Protein Kinase B
PMSCs	Placental Mesenchymal Stem Cells
POF	Premature Ovarian Failure
POI	Premature Ovarian Insufficiency
PPARγ	Peroxisome Proliferator-Activated Receptor gamma
PR	Progesterone Receptor
PRKAA2	Protein Kinase AMP-Activated Catalytic Subunit Alpha 2
RANTES	Regulated on Activation, Normal T Cell Expressed and Secreted
Rap1	Ras-proximate-1 or Ras-related protein 1
SCs	Stem Cells
SMAC	Second Mitochondria-derived Activator of Caspases
Smad	Small Mothers Against Decapentaplegic
SPECT	Single-Photon Emission-Computed Tomography
SSCs	Spermatogonial Stem Cells
STAT3	Signal Transducer and Activator of Transcription 3
TGFβ	Transforming Growth Factor beta
Th1	T helper 1 cell
Th17	T helper 17 cell
Th2	T helper 2 cell
TNFR1	Tumor Necrosis Factor Receptor 1
TNFα	Tumor Necrosis Factor alpha
Treg	Regulatory T cell
UC-MSCs	Umbilical Cord Mesenchymal Stem Cells
VEGF	Vascular Endothelial Growth Factor
VEGFR	Vascular Endothelial Growth Factor Receptor
Wnt5a	Wingless-Type MMTV Integration Site Family, Member 5A
